# Improving the Explosive Performance of Aluminum Nanoparticles with Aluminum Iodate Hexahydrate (AIH)

**DOI:** 10.1038/s41598-018-26390-9

**Published:** 2018-05-23

**Authors:** Jennifer L. Gottfried, Dylan K. Smith, Chi-Chin Wu, Michelle L. Pantoya

**Affiliations:** 1 Department of Mechanical Engineering, Texas Tech University, Lubbock, TX 79409 USA; 2 Weapons and Materials Research Directorate, U.S. Army Research Laboratory, Aberdeen Proving Ground, Aberdeen, MD 21005 USA

## Abstract

A new synthesis approach for aluminum particles enables an aluminum core to be passivated by an oxidizing salt: aluminum iodate hexahydrate (AIH). Transmission electron microscopy (TEM) images show that AIH replaces the Al_2_O_3_ passivation layer on Al particles that limits Al oxidation. The new core-shell particle reactivity was characterized using laser-induced air shock from energetic materials (LASEM) and results for two different Al-AIH core-shell samples that vary in the AIH concentration demonstrate their potential use for explosive enhancement on both fast (detonation velocity) and slow (blast effects) timescales. Estimates of the detonation velocity for TNT-AIH composites suggest an enhancement of up to 30% may be achievable over pure TNT detonation velocities. Replacement of Al_2_O_3_ with AIH allows Al to react on similar timescales as detonation waves. The AIH mixtures tested here have relatively low concentrations of AIH (15 wt. % and 6 wt. %) compared to previously reported samples (57.8 wt. %) and still increase TNT performance by up to 30%. Further optimization of AIH synthesis could result in additional increases in explosive performance.

## Introduction

The effect of aluminum (Al) additives on the laser-induced plasma chemistry of the military explosive cyclotrimethylenetrinitramine (RDX) was previously demonstrated using time-resolved emission spectroscopy^1^; increasing the Al content resulted in an increase in plasma temperature (due to the exothermic formation of AlO and Al_2_O_3_) and an increase in C_2_/soot formation because of the oxygen-scavenging Al reactions. Exothermic reactions were subsequently shown to increase the laser-induced shock wave velocity and/or the laser-induced deflagration reaction following pulsed laser excitation of energetic materials^2^. The strong correlation between the measured laser-induced shock velocities produced by laser ablation of energetic materials and the reported detonation velocities from large-scale detonation tests provides a laboratory-scale method for estimating the fast (i.e., microsecond-timescale) energy release from milligram quantities of energetic materials^3^. The laser-induced air shock from energetic materials (LASEM) technique has recently been used to estimate the detonation velocities of novel energetic materials^4,5^ and conventional military explosives doped with Al or boron (B) additives^6^. Slower energy release such as that from combustion reactions in air results in strong laser-induced deflagration reactions on the millisecond timescale^7^; typically, energetic materials such as trinitrotoluene (TNT) that make good propellants have significantly higher deflagration intensities than more powerful military explosives such as RDX that produce faster detonation waves (and thus, faster laser-induced shock velocities). Therefore, TNT is a good baseline explosive to evaluate the performance of metal particle additives that typically enhance late-time blast effects but inhibit detonation velocities.

Because of its high heat of oxidation, Al powder (typically 20% by weight) is commonly added to TNT to enhance the late-time blast effects of the explosion; tritonal is a typical Al-TNT formulation^8^. Since only a fraction of the available chemical energy is released from conventional metal particle formulations on the microsecond-timescale (resulting in lower detonation velocities), significant research efforts have been devoted to decreasing the reaction time of the metal additive in order to enhance the explosive performance^8^.

One approach recently introduced involves chemically altering the oxidation shell of an Al particle that is initially composed of an amorphous Al_2_O_3_ outer shell and crystalline Al core. When Al particles are exposed to oxygen during synthesis, an amorphous Al_2_O_3_ passivation layer forms around Al particles. The Al_2_O_3_ passivation layer can act as a heat sink and oxygen diffusion barrier that slows Al oxidation reactions. Capellos *et al*.^9^ and Baker *et al*.^10^ developed an eigenvalue detonation model to determine the effects of the Al_2_O_3_ passivation layer on Al oxidation when Al powders are used as additives in explosive mixtures. Their results show that the Al_2_O_3_ passivation layer surrounding Al particles delays the onset of Al oxidation such that Al oxidation does not occur in the detonation wave^9,10^. Reducing the limiting effects of the Al_2_O_3_ passivation layer could significantly decrease the ignition delay of Al particles and significantly enhance explosive performance. The motivation for this work is to explore chemical synthesis routes that alter the Al_2_O_3_ shell toward greater reactivity at microsecond timescales and are aligned with the chemical reactions behind the detonation wave.

When exposed to highly acidic solutions (i.e., pH < 4), Al_2_O_3_ becomes soluble and dissolves in solution^11^. The solubility of Al_2_O_3_ in acidic solutions has been utilized in the synthesis of Al energetic materials to remove the Al_2_O_3_ passivation layer surrounding Al particles and replace Al_2_O_3_ with a reactive salt^12,13^. In Smith *et al*.^12,13^, it was shown that aluminum iodate hexahydrate (AIH) replaces the Al_2_O_3_ passivation layer when Al particles are added to concentrated solutions of iodic acid. The Al^0^-AIH core-shell particles significantly increased reactivity with flame speeds measured as high as 3200 m/s^14^. These high flame speeds suggest the Al-AIH particles have great potential to exceed the reactive timescale limitations of Al particles when combined with explosives such as TNT.

The objective of this study is to examine the energy release from novel Al^0^-AIH samples that vary slightly in AIH concentration from ~6% AIH to 15% AIH (i.e., AIH6 and AIH15) then compare to the reactions of conventional micron-sized Al particles, alumina (Al_2_O_3_), and a physical mixture of Al/I_2_O_5_. This analysis is further extended to composites of TNT with each of these additives: micron-sized Al, Al_2_O_3_, Al/I_2_O_5_, AIH6 and AIH15. The goal is to understand AIH contribution to detonation and/or deflagration reactions using the laboratory-scale LASEM technique.

## Experimental

### Aluminum iodate hexahydrate synthesis and characterization

The mixing procedure used here is similar to the procedure used previously^12–14^. AIH mixtures were synthesized by first dissolving commercially available I_2_O_5_, supplied by Sigma Aldrich (St. Louis, MO), in distilled water prior to mixing 80 nm average diameter Al powder supplied by Novacentrix (Austin, TX). Both AIH6 and AIH15 were mixed to an equivalence ratio (ER) of 0.9 and the water to Al ratio for AIH6 and AIH15 was 1:1 and 2:1, respectively.

The XRD data for each sample was collected on a Rigaku Ultima III powder diffractometer operated in continuous θ-2θ mode from 15–60° 2θ with parallel beam geometry. The step size was 0.02° with a collection time of 2°/min. The MDI Jade V9.1.1 software provides both qualitative and quantitative data analysis. The uncertainty in the XRD measurements from peak fitting and variation between samples is calculated to be less than 6.9% for all concentrations.

The skeletal density of AIH6 and AIH15 were also measured using an AccuPyc II 1340 pycnometer from Micromeritics (Norcross, GA). For each sample, 15 volume measurements were run with a nitrogen flow of 0.005 psig/min. Samples were weighed prior to volume measurements and density was calculated from weight and volume measurements. The standard deviation from pycnometer measurements is less than 0.025 g/cm^3^. All of these measured densities are above 90% of the theoretical maximum density. The oxygen balance (*OB*) is calculated in terms of 100 grams of material to determine the percent of oxygen excess or deficient for 100 grams of a compound.

1 $$OB \% =\frac{-1600}{{M}_{c}}\ast (2X+\frac{Y}{2}+\alpha M-Z)$$

In Eq. (1), *M*_*c*_ is the molecular weight of the compound, *X* is moles of carbon (i.e., 0 for this mixture since carbon is not present), *Y* is moles of hydrogen, *αM* is moles of metallic oxide, and *Z* is the moles of oxygen.

The AIH6 and AIH15 samples were further examined using the transmission electron microscopy (TEM) technique. The TEM specimens were prepared using high purity ethanol (Decon Laboratories, Inc.) as the medium via the nanoparticle suspension technique^15^. The samples were studied in a JEOL 2100FX TEM operated at 200 keV (JEOL USA, Inc.). The overall field of view varied from 10 to 100 µm^2^ under TEM diffraction contrast imaging conditions with an average emission current approximately 120 µA. Fourier transform (FFT) patterns were acquired to reflect the crystallinity of the sample from corresponding TEM images. The surface chemistry of the particles was further analyzed using a Physical Electronics VersaProbe II Ultra X-ray Photospectroscopy system equipped with a hemispherical analyzer and a take-off angle of 45 degrees. The sample was irradiated by a focused beam of monochromatic Al Kα X-rays.

Differential scanning calorimetry (DSC) for one AIH sample was performed using a Netzsch Model STA 449 to measure the heat flow as a function of temperature and time. Only one sample was analyzed because the analysis resulted in thermal runaway and significant damage to the instrumentation. For the DSC analysis, a 7 mg sample of AIH6 was loaded into an alumina crucible with no lid and placed in the diagnostic. The sample was heated in an argon environment at 10 K/min (KPM). Temperature calibrations for the instrument were performed using melting of a set of metal standards resulting in a temperature accuracy of ±1 °C.

### Laser-Induced Air Shock from Energetic Materials (LASEM) Experiments

The LASEM experimental setup has been described previously^2,3^. Briefly, a 6-ns pulsed Nd:YAG laser (Quantel Brilliant b, 1064 nm, 900 mJ) is focused just below the sample surface with a 10 cm lens. The laser ablates the sample material into the air above the sample surface, atomizing/ionizing a fraction of the ejected material and forming a high-temperature (>10,000 K) microplasma. Because of the properties of the laser (pulse duration and wavelength), most of the energy of the laser pulse is absorbed by the plasma, which shields the sample surface. During the plasma lifetime (10’s of μs), the ablated material is highly excited, resulting in strong atomic and molecular emission spectra. The plasma rapidly cools following the laser pulse, and recombination reactions occur which result in the formation of molecular species and, if the reactions are exothermic, increase the plasma temperature. While even inert samples produce strong laser-induced shock waves due to the energy deposited by the laser in the plasma, the chemical reactions of energetic materials further increase the plasma temperature and resulting shock wave velocity. The expansion of the shock wave into the air above the sample is visualized using a typical Z-type schlieren imaging setup (10.8 cm diameter mirrors, 114 cm focal length) illuminated by a 200 W Hg-Xe arc lamp. A high-speed color camera (Photron SA5) with a zoom lens (Nikon Nikkor 24–85 mm f/2.8-4D IF) records the shock wave expansion at 84,000 frames-per-second (64 × 648 pixels, 1.0 μs shutter). The measured shock wave positions are then used to plot the laser-induced shock wave velocity as a function of time. The resulting data is fit to a polynomial; the y-intercept of the fit is the characteristic shock velocity for the sample under the given experimental conditions and is used to compare the fast energy release of the materials. For energetic materials, the measured characteristic shock velocity can be used to estimate the detonation velocity of the sample (at the theoretical maximum density) using the previously determined calibration fit^3^. Additional diagnostics for the LASEM setup used in this study included a CCD spectrometer (Ocean Optics USB4000, 200–890 nm, 100-ms integration time) to investigate the chemical reactions of the laser excited material and an infrared-sensitive photodiode (New Focus Model 2053, 900–1700 nm, 10^2^ gain) to measure the extent (i.e., peak emission intensity and duration) of the combustion reactions. An exhaust outlet was located next to the laser ablation region to remove scattered particulates and product gases.

As with previous LASEM experiments, the sample substrate was double-sided tape on a glass microscope slide. Residues of varying thicknesses (8–63 μg/mm^2^) were prepared on multiple slides for each sample (except for Al_2_O_3_) by pressing the sample firmly into the tape with a metal spatula (to confine the material in the laser focus and enhance the laser-material interaction). At least 20 laser shots were acquired for each sample. The sample slides were weighed before and after each laser shot with a balance accurate to 1 μg in order to determine the amount of material removed with each laser shot (via ablation, chemical reaction into gaseous products, or ejection off the sample surface). Pure metal samples investigated included micron-sized Al (Sigma-Aldrich, 27.5 μm mean particle size), reduction-grade alumina (Al_2_O_3_, NIST SRM 699), Al/I_2_O_5_ (80 nm Al, 0.9 equivalence ratio), AIH6, and AIH15. Prior to the LASEM experiments, the AIH6 sample vial contained iodine vapor, indicating some sample degradation had occurred (Fig. S1). The AIH15 vial remained iodine-free for approximately one week following the LASEM data collection. The metals were added to TNT (provided by colleagues at the U.S. Army Research Laboratory) in composite mixtures that were approximately 20% metal by weight.

## Results

### AIH Characterization

Table 1 shows the density and composition of AIH6 and AIH15 determined by Powder XRD analysis. OB is calculated from concentration data using Eq. 1. The composition of a similar mixture (AIH ER 0.9) from previous work is shown for comparison. The mixing procedure used here is similar to the procedure used previously^12–14^; however, the final composition of the AIH6 and AIH15 are significantly different as shown in Table 1. The concentrations of Al°, AIH and iodic acid from AIH6, AIH15 and another AIH sample mixed at an initial ER of 0.9 discussed in Smith *et al*.^13,14^ are included in Table 1 for comparison. The only difference between the AIH samples in Table 1 is the initial water to Al ratio used during mixing. The differences in concentrations of AIH between the three samples shown in Table 1 indicate that there are many unknown variables involved in AIH synthesis that need to be studied further, including drying time and solution pH.

**Table 1:** AIH-Al mixture composition.

Sample	Water to Al ratio	AIH (wt. %)	Al° (wt. %)	β-HIO_3_ (wt. %)	HI_3_O_8_ (wt. %)	Density	OB
AIH6	1:1	5.8	19.2	75.0	0	3.98	1.6%
AIH15	2:1	15.0	20.0	52.4	12.6	4.36	0.4%
AIH ER 0.9^12–14^	2.4:1	57.8	18.6	11.9	11.6	3.39	0.3%

**Figure 1:**
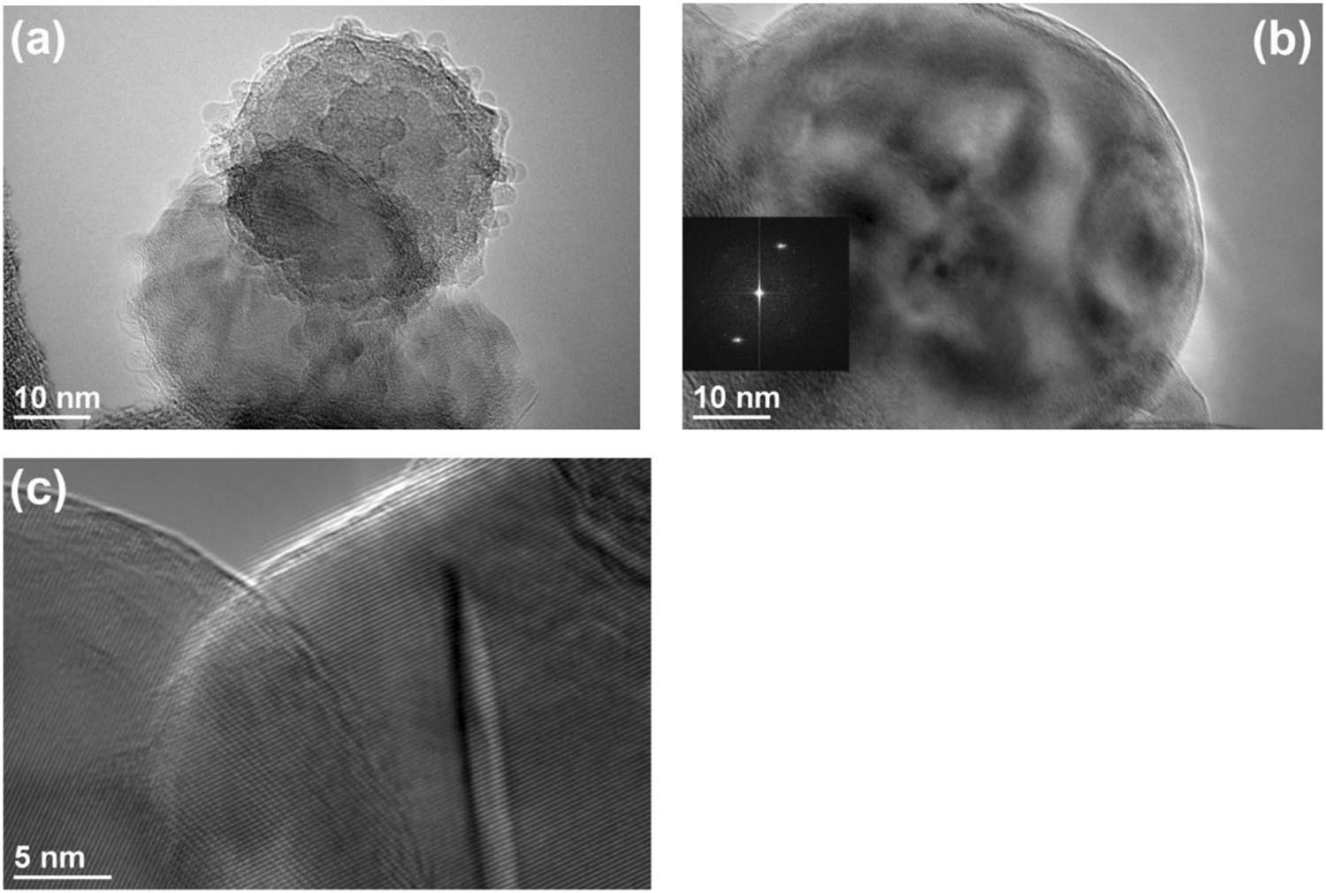
TEM images showing the nature of Al-AIH nanoparticles: (**a**) the rough surface with protruding nodules, (**b**) and (**c**) the single crystallinity exemplified by the distinct lattice fringes showing as parallel thin straight lines and the different strain contrast due to the AIH layer in 300 kx and 800 kx magnifications, respectively.

Figure 1 illustrates the representative nature of the resultant Al-AIH nanoparticles through high resolution TEM (HRTEM) images. Figure 1a shows that the AIH layer is exhibited as nodules protruding from the Al surface and enhancing the roughness. Figure 1b shows a large Al-AIH nanoparticle and its single crystallinity was exemplified by the lattice fringes shown as parallel thin straight lines in the TEM image and the corresponding diffraction spots in the FFT pattern. Figure 1c is an image at 800 kx magnification to highlight the single crystallinity of the Al-AIH nanoparticle and the different diffraction contrast due to the presence of the AIH phase.

**Figure 2:**
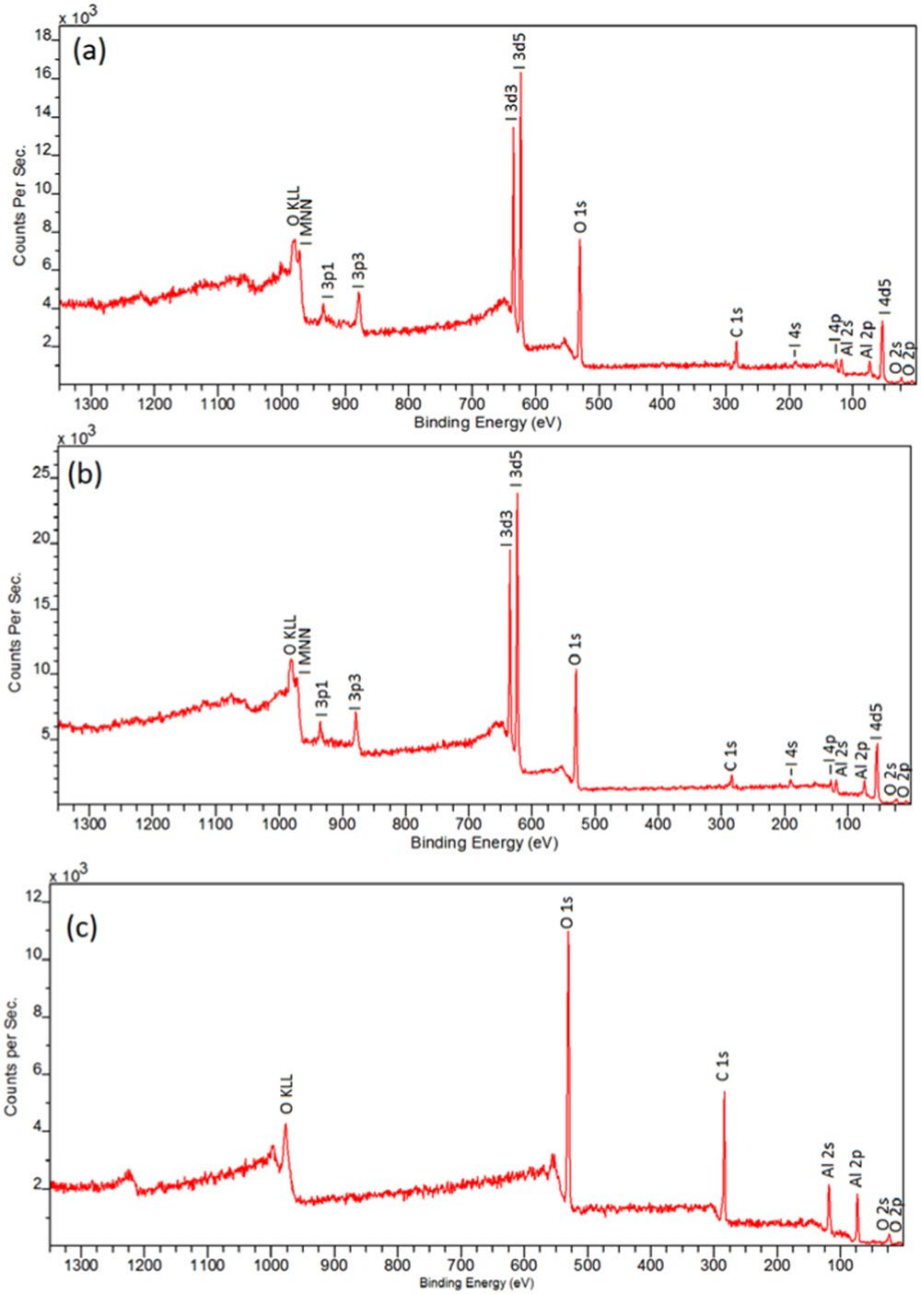
XPS surveys confirming the presence of iodine on the surface of the (**a**) AIH6, (**b**) AIH15 particles, in comparison to (**c**) untreated Al particles.

The presence of iodine on the AIH6 and AIH15 samples is further confirmed via XPS analysis. Shown in Fig. 2a,b are the binding states of these samples displaying all signature iodine peaks at binding energies of ~ 49 eV, 121 eV, 186 eV, 619 eV, 631 eV, 875 eV, and 931 eV, along with Auger signals, which do not exist in uncoated Al particles (Fig. 2c).

### Laser-Induced Shock Waves

Shot-to-shot variations in the laser-material interaction are well-known to influence ablation mass and laser-induced plasma properties^16–18^, particularly for residue samples^19^. Minimizing these variations involves control of the experimental parameters, obtaining sufficient data points for statistical averaging, and/or analytical methods to compensate for signal fluctuations^20,21^. In this work, the first two methods were used to decrease the shot-to-shot variations as much as possible – however, it was not possible to completely overcome the stochastic nature of the laser-material interaction. Moreover, quantifying the shot-to-shot variations is challenging because of all the different processes involved in the laser-material interaction. For example, although we can measure the amount of material removed after each laser shot, it is not possible to identify where the material went, i.e., whether it was ablated into the plasma where it participated in the chemical reactions, or it was ejected off the sample slide and subsequently reacted in either the plasma region or (later) the deflagration zone, or it was ejected off the sample slide away from the interaction region. Of the material ablated into the plasma, only some fraction of it is completely atomized. The largest amount of material is ejected off the sample slide during the first several laser shots, as the shock wave disperses the loose material on the slide (Fig. S2). Table 2 shows the wt.% of metal, residue thickness, average mass removal per laser shot, and number of laser shots for each sample Typically, the first several laser shots removed the most material from the slide (up to 10× the mass of subsequent shots) since the shock wave disperses the loose material on the slide.

**Table 2:** Sample details and number of laser shots acquired.

Sample	%Metal by weight	Residue thicknesses (μg/mm^2^)	Average mass removal per laser shot (mg)	#Laser shots
Al	100	37.8–63.1	0.44 ± 0.60	23
Al_2_O_3_	100	10.0	0.26 ± 0.37	20
Al/I_2_O_5_	100	1.92–7.56	0.15 ± 0.23	22
AIH6	100	18.7–28.4	0.40 ± 0.49	22
AIH15	100	7.94–24.3	0.23 ± 0.28	24
TNT	0	29.8–62.3	1.04 ± 0.81	20
TNT-Al	23	19.7–38.6	0.68 ± 0.69	25
TNT-Al_2_O_3_	23	27.2–45.2	1.02 ± 0.53	21
TNT-Al/I_2_O_5_	20	11.5–32.9	0.54 ± 0.57	22
TNT-AIH6	23	24.6–43.9	0.76 ± 0.59	22
TNT-AIH15	21	19.0–39.4	0.68 ± 0.53	22

For most samples, the amount of material removed from the substrate reaches a steady state around 100 μg per shot; in general, the deflagrating samples (TNT composites) result in more material removal per shot (Table 2). For a few samples (e.g., AIH15, TNT), a jump in the amount of mass removed at later shot numbers results from a pile of material created by a previous shock wave igniting.

**Figure 3:**
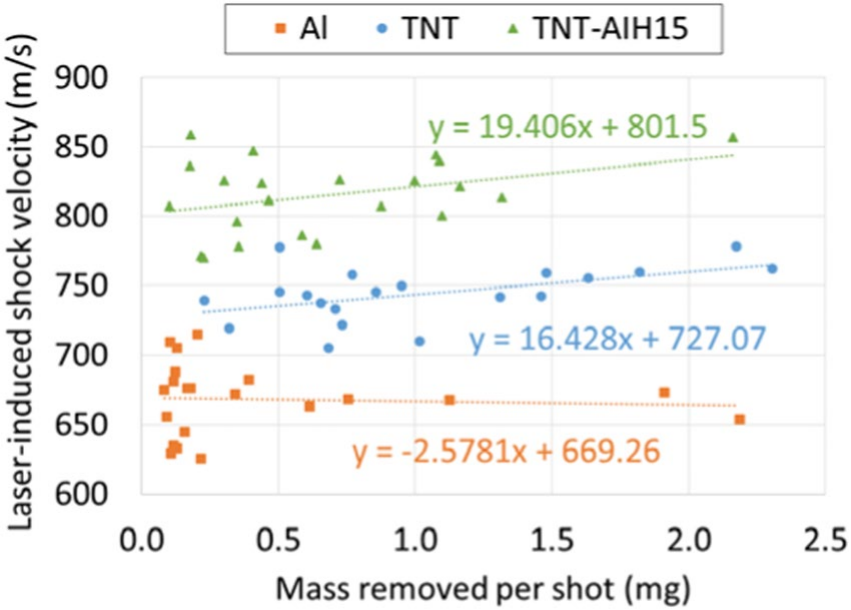
Laser-induced shock velocities for Al, TNT, and TNT + AIH15 as a function of the amount of material removed from the sample slide per laser shot.

We investigated the effect of the amount of material removed per laser shot on the measured laser-induced shock velocities (Fig. 3). Although some of the materials showed a very slight correlation between the mass removed per shot and the measured shock velocity, in general the amount of material removed from the slide did not significantly influence the characteristic shock velocity. Since the largest sources of error in the shock velocity measurement are the shot-to-shot variations in the laser-material interaction and the measurement errors in the shock wave position (and subsequent y-intercept determination), the slight effect of residue thickness is within the error bars of the LASEM measurement. This is because the amount of material in the laser focus is relatively fixed by the defined focal length and the amount of material held in place by the tape. Any excess material is ejected off the sample surface and reacts (if at all) on the millisecond timescale, not on the microsecond timescale that determines the shock velocity.

**Figure 4:**
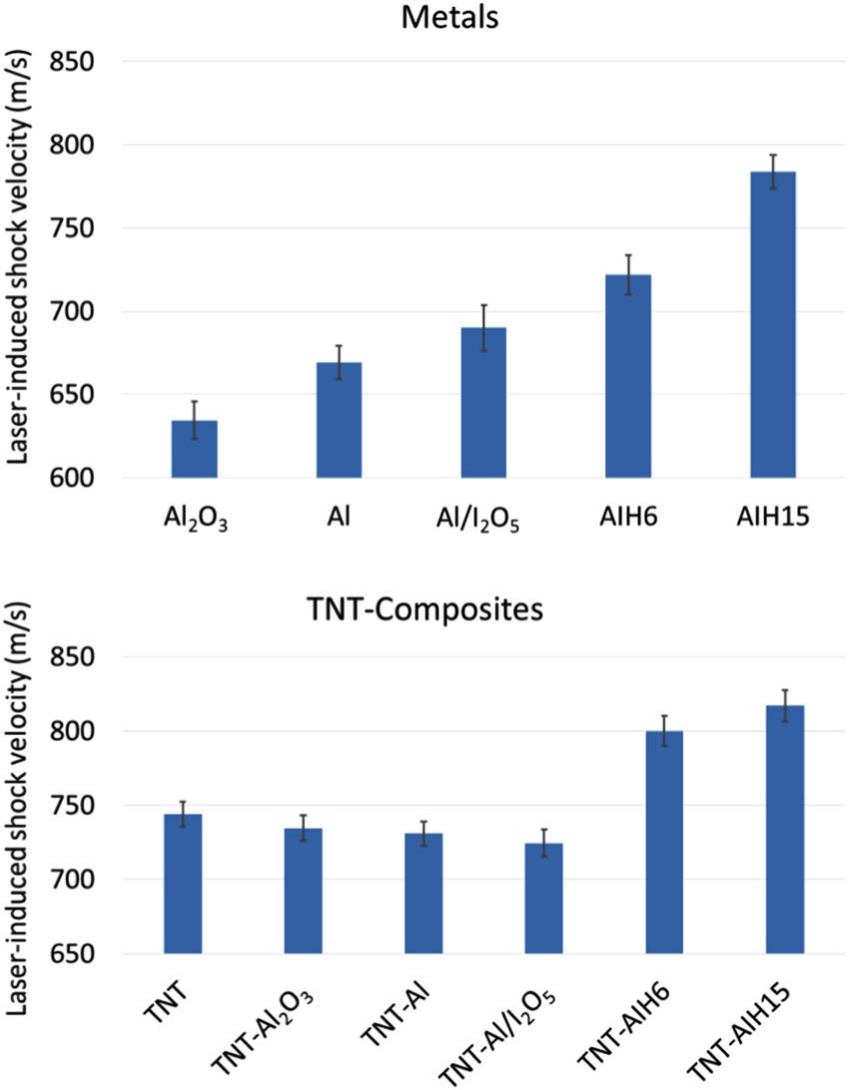
Laser-induced shock velocities for the metal additives (top) and TNT composites (bottom).

Figure 4 shows the average characteristic laser-induced shock velocities measured for the pure metal additives (top) and TNT composite (bottom) samples. The Al_2_O_3_ has the lowest energy release because relatively little Al is free to oxidize in air on the microsecond timescale. While the physical Al/I_2_O_5_ mixture released more energy than the micron-sized Al, the AIH samples released the most energy on the microsecond timescale, with the AIH15 sample (which showed no visible signs of degradation) producing the highest shock velocity. It is not clear that the measured laser-induced shock velocities for the AIH samples would directly correlate to detonation velocities (as with the primarily organic military explosives); however, the significant increase in measured laser-induced shock velocities for the AIH samples compared to the other metal additives suggest that they release their energy on a much faster timescale relevant to explosive events.

To test this hypothesis, the laser-induced shock velocities for TNT composites with the additives were also measured (bottom of Fig. 4). As previously shown, the micron-sized Al decreases the laser-induced shock velocity (and in large-scale tests, the detonation velocity), in part due to the formation of solid reaction products^6^. The Al_2_O_3_ additive also decreases the shock velocity, although to a somewhat lessor extent since fewer solid reaction products form during the first few microseconds of the high-temperature reactions to slow the shock wave (compared to micron-sized Al). The biggest decrease in shock velocity was observed for the TNT-Al/I_2_O_5_ composite. In contrast, the TNT-AIH samples resulted in a significant increase in laser-induced shock velocities. Using the calibration fit determined for military explosives^3^, the estimated detonation velocities for the TNT-AIH6 and TNT-AIH15 composite materials are 8.69 ± 0.24 km/s and 9.10 ± 0.26 km/s, respectively (compared to an estimated TNT detonation velocity^3^ of 7.03 ± 0.12 km/s).

**Figure 5:**
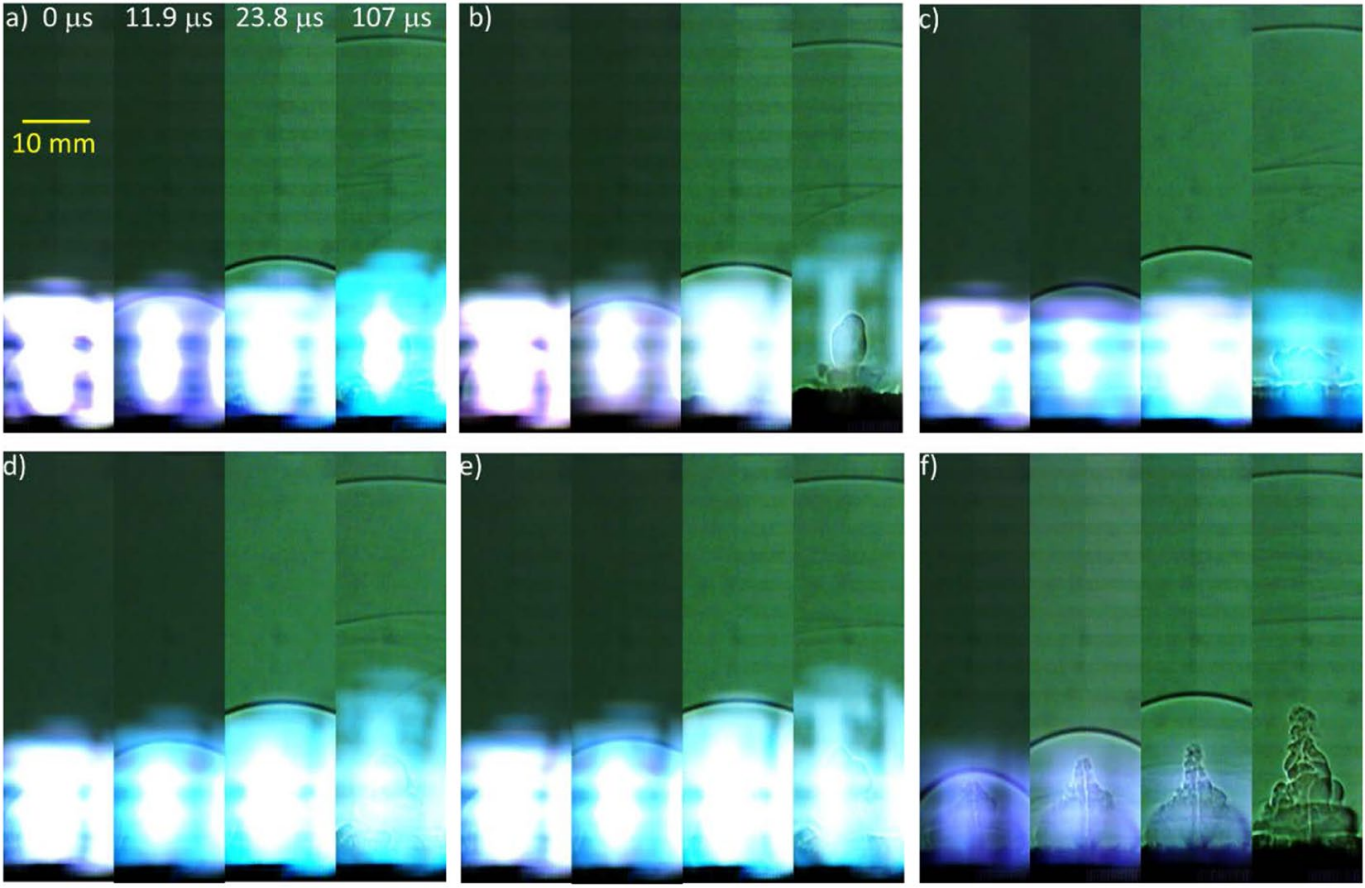
Snapshots from videos of laser excited (**a**) micron-sized Al, (**b**) Al_2_O_3_, (**c**) Al/I_2_O_5_, (**d**) AIH6, (**e**) AIH15, and (**f**) TNT.

**Figure 6:**
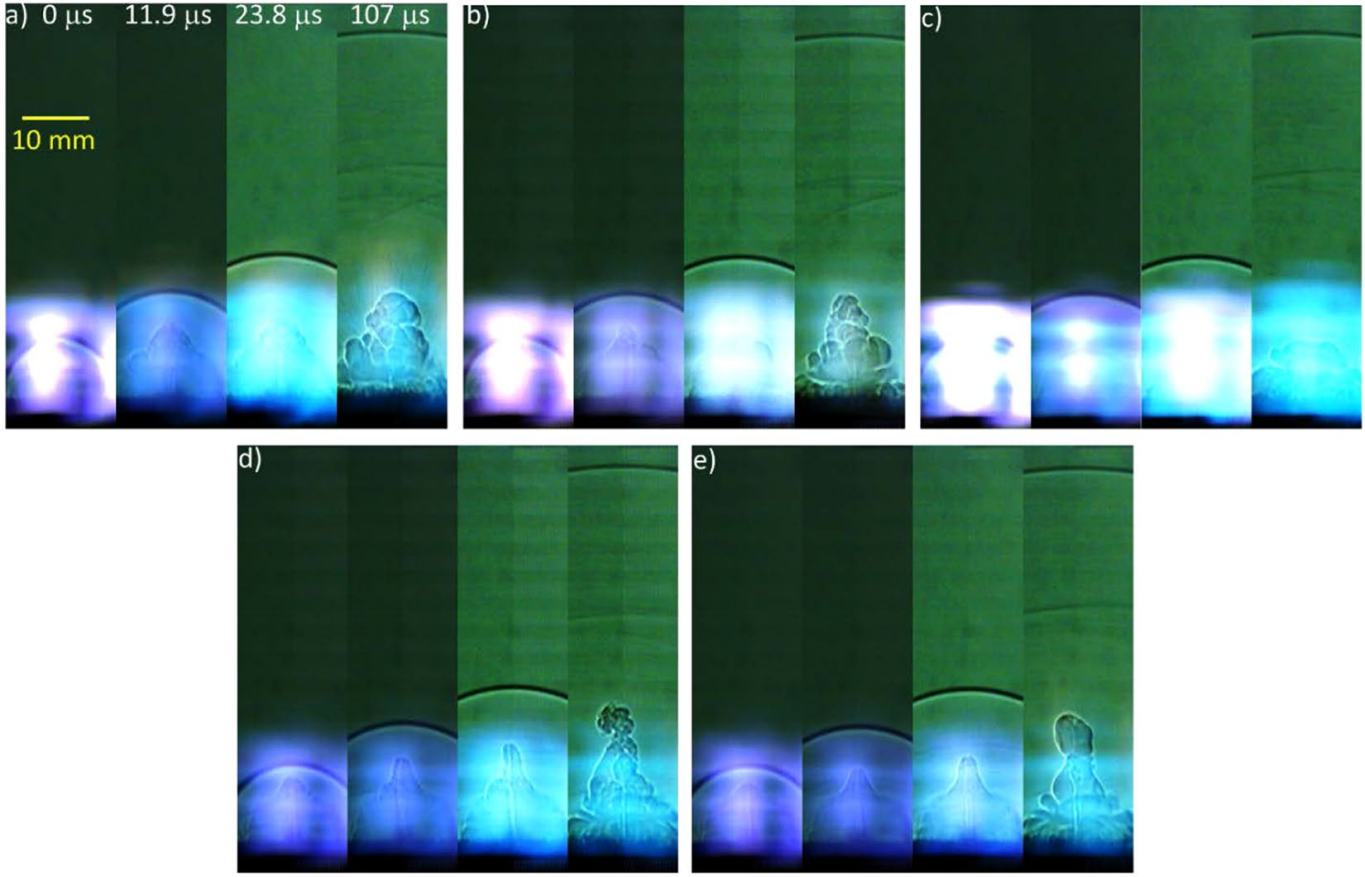
Snapshots from videos of laser excited (**a**) TNT + Al, (**b**) TNT + Al_2_O_3_, (**c**) TNT + Al/I_2_O_5_, (**d**) TNT + AIH6, and (**e**) TNT + AIH15.

Snapshots from the high-speed videos of the laser excitation of the pure and composite samples are shown in Figs 5 and 6, respectively. The brightness and contrast of some of the later frames have been adjusted to improve visualization of the shock wave, and the top of the images have been cropped. The metal additives produce brighter plasmas as a result of the extensive aluminum-related emission features while energetic materials such as TNT produce less intense plasmas, as previously observed^2,6^. The reduced plasma emission from the TNT-AIH composites (compared to TNT with the other Al-containing additives) is indicative of increased energy release; the reduced emission is likely a result of the formation of exothermic reaction products which do not emit in the visible wavelength region (unlike neutral or ionic Al).

### Laser-Induced Deflagrations

While the pure micron-sized Al sample resulted in a significant combustion cloud above the sample surface following laser excitation (lasting approximately 40 milliseconds on average, Fig. S3), neither the Al_2_O_3_ nor the Al/I_2_O_5_ mixture resulted in significant reaction following the decay of the laser-induced plasma. The combustion of the Al particles ejected into the air above the sample surface was similar to that previously observed during the laser-induced deflagration of energetic materials^7^. Although the Al_2_O_3_ and Al/I_2_O_5_ residues were not as thick as the micron-sized Al because of the way the material spread on the tape, shots with similar amounts of material removal (~100 μg per shot) could be compared directly to account for the differences in excess material; even with comparable masses of material ejected above the sample surface, of these three samples only the micron-Al sample resulted in late-time combustion emission. Although no cloud of combusting particles was produced in the air directly above the focused laser position following laser excitation, the AIH samples were more sensitive to the burning metal particles produced by the laser ignition than the other Al-containing additives investigated here. For both the pure AIH6 and AIH15 samples, the reaction following laser excitation propagated to other areas on the sample slide for two of the laser shots (out of 22 and 24, respectively), as shown in Fig. S4. In each case, additional material on the sample slide ignited several milliseconds after the laser pulse. The ignition of adjacent material only occurred for the first two laser shots on the affected sample slides, suggesting that the reaction involved excess material (subsequently ejected off the sample slide by successive laser-induced shock waves). The ignited material was located several centimeters distant from the location of the focused laser pulse and was most likely ignited by burning metal particles ejected from the initial reaction zone. No reaction propagation to subsequent areas of the sample slide was observed for the micron-sized Al, Al_2_O_3_, or Al/I_2_O_5_ samples, even when excess material was present, suggesting that the AIH samples are more sensitive than the other Al-containing materials.

**Figure 7:**
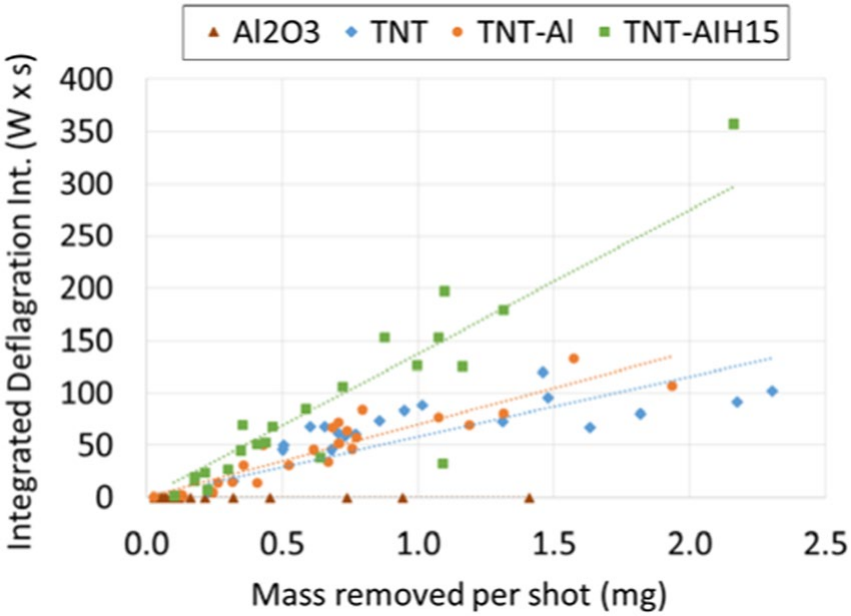
Integrated deflagration emission for Al_2_O_3_, TNT, TNT-Al and TNT-AIH15 as a function of the amount of material removed from the sample slide per laser shot.

**Figure 8:**
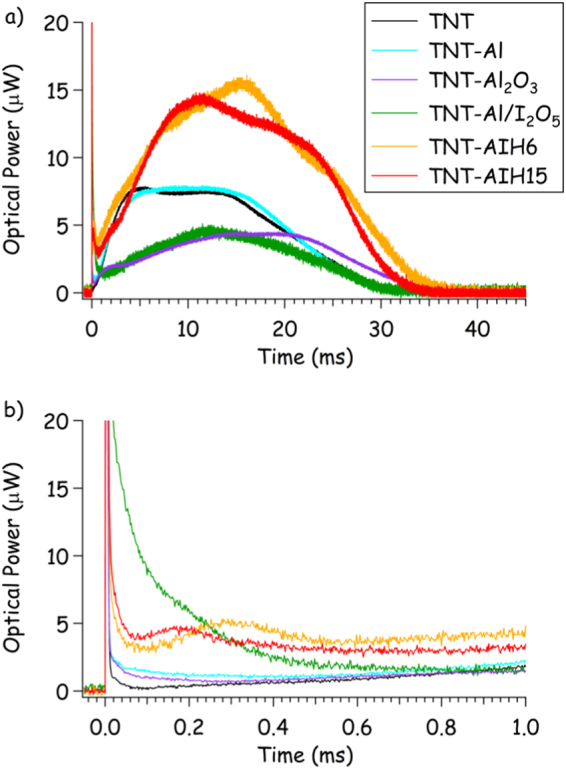
Average time-resolved emission from the laser-induced (**a**) deflagration and (**b**) microsecond-timescale combustion of TNT composites (limited averages from shots with 1.0 ± 0.2 mg of material removed).

The intensity and duration of the laser-induced deflagration increases with the amount of material ejected into the air above the substrate (for those materials that deflagrate). Thicker residues generally result in more material ejected per laser shot and thus stronger deflagration events on the millisecond timescale (Fig. 7). The average laser-induced deflagration emission intensities (measured in units of optical power by the photodiode) for the TNT composites (Fig. S5) show that the TNT-Al formulation produces the most intense deflagration events, followed by pure TNT, as previously observed^6^. The TNT-Al_2_O_3_ and TNT-AIH composites produced comparatively weaker deflagrations, with the TNT-Al/I_2_O_5_ producing the weakest deflagration. For comparison to the average deflagration intensities over all laser shots (Fig. S5), limited averages of the deflagration intensities were calculated using only the laser shots where 1.0 ± 0.2 mg of material was removed from the slide (Fig. 8). Although the measured mass removal amount is not necessarily indicative of how much material was available in the deflagration zone, limiting the number of averaged shots in this way could partially correct for the amount of material ejected from the sample surface. As shown in Fig. 8a, the limited averages suggest that when similar amounts of material are available to react with the heated air above the sample surface, the AIH samples produce the strongest deflagration reactions. The differences between the average and limited average deflagration intensities could therefore reflect differences in the adhesion of the material to the tape, and not just the material’s potential for extended combustion reactions in air. Unlike with the pure AIH samples, no reaction propagation to adjacent spots on the sample slide was observed for the TNT-AIH mixtures (although, in general, significantly more material is ejected from the sample slide, Table 2).

The emission spectra of the aluminum additives have similar features (Fig. S6). Strong emission features due to Al, AlO, CN and Na (a common impurity) saturated the spectrometer for some samples. The most surprising feature is the strong CN molecular bands, since the metal additives likely do not contain much carbon. Although carbon-related features from the tape used to adhere the residue to the substrate typically are not very strong in the resulting emission spectra, the presence of the oxygen-scavenging Al may enhance the formation of CN through recombination of the available carbon with nitrogen from the air rather than oxygen (to form CO, CO_2_)^1^. Unfortunately, the strong AlO molecular emission bands obscure the C_2_ emission features (512 and 516 nm) which could help confirm this hypothesis (since a lack of oxygen available for reaction with the C would result in increased C_2_ emission^1^). Among the aluminum-containing additives, Al/I_2_O_5_ has the strongest AlO bands, while micron-sized Al has the strongest CN and H emission features. Al_2_O_3_ has the most Na contamination, along with Li and K. AIH6 has Mg and Ca contaminants. While the Al emission features are significant for all Al-containing materials, atomic I features are comparatively weak and do not appear in the spectra. No evidence of possible reaction intermediates such as the iodine monoxide radical (IO; 427.2, 449.3, and 484.9 nm), iodine oxide (OIO; 549.3 nm), or I_2_ (500 nm continuum)^22^ was observed, although any emission features present were likely obscured by the strong AlO bands. The TNT and TNT composite material spectra (Fig. S7) have similar emission features as the pure metal additives, along with a broad emission feature in the visible region due to grey body radiation from the deflagration of the material following laser excitation.

## Discussion

### Microsecond-Timescale Reactions

Figures 3 and 4 show that AIH increases the laser-induced shock velocities of TNT. The estimated detonation velocity of the TNT-AIH15 was 9.10 ± 0.26 km/s, indicating AIH15 may increase the detonation velocity of TNT by up to 30%. The increase in estimated detonation velocity in TNT-AIH15 mixtures suggests that the reaction time of AIH is on a fast enough time scale (μs) that significant amounts of gaseous products (I_2_, O_2_, aluminum iodates species, etc.) could be formed in the chemical reaction zone behind the detonation wave. While similar products are formed from the thermite reaction of Al with I_2_O_5_, they are not produced on a fast-enough timescale to increase the shock velocity, as evidenced by the decrease in laser-induced shock velocity for the TNT-Al/I_2_O_5_ composites. In Capellos *et al*.^9^ and Baker *et al*.^10^, it was shown that the Al_2_O_3_ passivation layer limits Al reactions and slows the detonation velocity of aluminized explosives. Figure 8b shows that as the laser induced plasma is rapidly cooling (i.e., as the plasma emission decreases within the first 50 μs), extended emission from combustion of the TNT-Al/I_2_O_5_ composites continues for approximately 500 μs. These reactions (beyond approximately 10 μs) are too slow to contribute to the laser induced shock velocity. The increase in laser-induced shock velocity of TNT-AIH15 mixtures suggests that the limiting effects of the Al_2_O_3_ passivation layer are reduced in AIH formulations. In Smith *et al*.^13,14^, it was shown that during synthesis of AIH, the Al_2_O_3_ passivation layer is dissolved and replaced with AIH. The increase in estimated detonation velocity seen in TNT-AIH15 mixtures suggests that when the Al_2_O_3_ passivation layer is replaced with AIH, the Al-AIH reaction occurs on a timescale relevant to detonation. In addition, Fig. 8b also shows peaks in the combustion emission near 180 and 300 μs for AIH15 and AIH6, respectively. These combustion reactions are likely ignited by the plasma and, unlike the subsequent deflagration reactions (on the millisecond timescale, Fig. 8a), are not self-sustaining.

**Figure 9:**
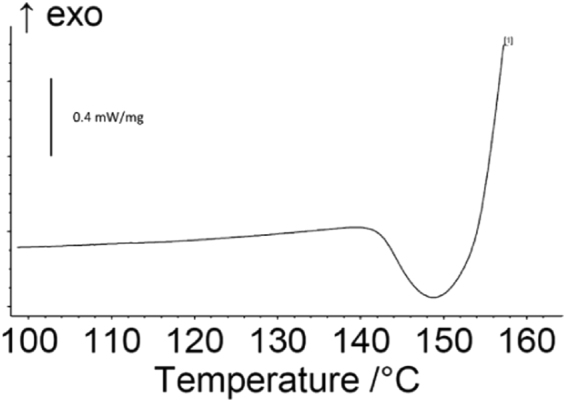
Heat flow of AIH6 heated at 10 °C/min in an argon atmosphere. Scale bar corresponds with 0.4 mW/mg.

In Smith *et al*.^14^, it was also proposed that AIH reactions occur when the hydrate layer in AIH is removed. In Cradwick *et al*.^11^, the evaporation of the hydrate layer in AIH is reported to occur at 135 °C. To test the hypothesis that Al oxidation in AIH mixtures occur when the hydrate layer is removed, a DSC experiment was performed to examine heat flow under equilibrium conditions. Figure 9 shows the DSC heat flow of AIH when heated at 10 °C/min in an argon atmosphere. The heat flow plot shows an endotherm at 140 °C followed by a runaway exothermic reaction, confirming that AIH reactions are initiated by removal of the hydration layer in AIH. The heat flow ends between 150 °C and 160° because at this point, the thermal runaway of the AIH reaction damaged the DSC thermocouple to a point where measurements could not be taken. The temperature required to evaporate the hydrate layer in AIH is exceeded within the first nanosecond of the laser-material interaction as the laser-induced plasma is formed. Removal of the hydrate layer to initiate the reaction may contribute to the AIH samples reacting on times scales relevant to a detonation.

It is important to note that for this measurement, 7 mg of AIH mixture was used and the runaway reaction destroyed the sample and reference crucible, the platinum thermocouple, and ~6 cm of the thermocouple support. Use caution when preforming DSC experiments with AIH mixtures.

### Millisecond-Timescale Reactions

We have shown that removal of the hydration layer causes AIH to react on microsecond timescales, producing gaseous products formed in the reaction zone behind the laser-induced shock wave (Fig. 4). Figure 8a shows that TNT-AIH not only reacts on microsecond timescales, but continues to deflagrate on a millisecond timescale. The initial spike in emission intensity shown in the photodiode traces is due to the laser-induced plasma emission; ignition of the material ejected into the air above the sample occurs following the passage of the laser-induced shock wave – for the TNT composites, this is sufficient to initiate self-sustaining laser-induced deflagration reactions (i.e., fast combustion) on the millisecond timescale. The TNT-Al_2_O_3_ composite deflagration intensity peaked almost 10 ms later than the other composites, possibly indicative of the time required to free the Al content for further oxidation in air. The strong deflagrations of the TNT-AIH composites demonstrate additional energy release through combustion reactions on the millisecond timescale (compared to the other TNT composites with comparable amounts of ejected material). These results suggest that, in addition to improving the detonation performance, AIH could also potentially enhance the middle- and late-time blast effects (i.e., slow energy release).

### Mechanisms for Energy Release

Figure 4 shows that AIH reacts on a microsecond timescale and Fig. 8a shows that deflagration continues on a millisecond timescale. Reactions on a microsecond timescale suggest two possible mechanisms for AIH contributing to the TNT shock velocity. First, the plasma temperature could be increased for the TNT-AIH composites because more exothermic reactions are occurring. While the temperature of a laser-induced plasma formed on an inert substrate rapidly decays following cessation of the laser pulse (reaching just a few thousand Kelvin within several microseconds), exothermic reactions from ablated material can contribute additional energy to the plasma, raising the temperature and increasing the shock velocity beyond that observed using the same laser energy on an inert material. Significant increases in plasma temperature with the addition of Al nanoparticles to RDX have been previously observed^1^. The larger temperature differential between the plasma and the surrounding air would produce a faster shock. Second, Fig. 9 suggests that AIH produces gas products when the hydration layer is removed (Eq. 2) and corresponds with early stages of the reaction.

2 $$({[Al{({H}_{2}O)}_{6}]}^{3+}{(I{O}_{3})}_{3}^{-}{(HI{O}_{3})}_{2})\to {(A{l}^{3+}{(I{O}_{3})}_{3}^{-}{(HI{O}_{3})}_{2})}_{s}+{[{({H}_{2}O)}_{6}]}_{v}$$

Water vapor from dehydration production may contribute to accelerating the shock wave on the microsecond time scale. When the hydration layer is removed, one possible intermediate reaction could include the formation of iodine and oxygen, shown in Eq. 3.

3 $$2{(A{l}^{3+}{(I{O}_{3})}_{3}^{-})}_{s}\to A{l}_{2}{O}_{3}+{(\frac{15}{2}{O}_{2})}_{v}+{(6{I}_{2})}_{v}$$

Oxygen and iodine gas products from Eq. 3 may also contribute to accelerating the shock wave on the microsecond time scale. Only radical (or ionized) species are shown in Eq. 3. The stable product species (e.g., HIO_3_ from Eq. 1) that are not shown in Eq. 3 could enhance deflagration.

The enhancements in deflagration that occur after AIH has reacted behind the laser-induced shock wave could be a result of intermediate species reacting with oxygen from the air and/or HIO_3_ and HI_3_O_8_ reacting with the remaining Al°. In both Fig. S5 and Fig. 8a, the deflagration intensity for the TNT-AIH mixtures is greater than the TNT-I_2_O_5_ mixtures. If deflagration of the AIH mixtures is a result of the remaining HIO_3_ and HI_3_O_8_ reacting with Al°, similar deflagration intensities would be expected between the TNT-AIH mixtures and the TNT-Al/I_2_O_5_ mixtures; however, the Al_2_O_3_ shell in the TNT-Al/I_2_O_5_ mixtures is still intact. Since the deflagration intensities for the TNT-AIH mixtures are greater than the TNT-Al/I_2_O_5_ mixtures, dissolution of the Al_2_O_3_ shell not only allows reactions to occur on the microsecond timescale, but also results in more complete Al° oxidation during deflagration. This is supported by comparing the deflagration intensities of the TNT-AIH6 and TNT-AIH15 mixtures. The iodic acid concentration (both HIO_3_ and HI_3_O_8_) for the AIH6 mixture is 10.0 wt. % greater than the AIH15 mixture and the deflagration intensity (Fig. 8a) for AIH6 is greater than for AIH15. Higher concentrations of HIO_3_ and HI_3_O_8_ result in higher intensity deflagrations for the AIH6 mixture compared to the AIH15 mixture. Higher concentrations of AIH in AIH15 produce more vapor products in the intermediate reactions and result in higher laser-induced shock wave velocities.

Figure 4 shows that replacement of the Al_2_O_3_ passivation layer with AIH can significantly increase explosive performance. From Table 1, the AIH15 (15.0 wt. % AIH) mixture has a significantly greater concentration of AIH compared to AIH6 (5.8 wt. %). The variables that affect the final concentrations of AIH mixtures are still largely unknown; however, it was shown in Smith *et al*.^14^ that reactivity from AIH mixtures is directly related to concentration of AIH. In comparison to previous samples tested^13,14^ (with AIH concentration as high as 78%), only a fraction of the Al_2_O_3_ shell was replaced with AIH in AIH6 (5.8 wt. %) and AIH15 (15.0 wt. %) and as much as a 30% increase in estimated detonation velocity was seen in TNT-AIH mixtures. Higher concentrations of AIH in the AIH15 sample may account for the faster laser-induced shock velocities for TNT-AIH15 compared to TNT-AIH6. In addition, the AIH6 sample showed visible signs of degradation at the time of testing, while the AIH15 sample did not. In general, AIH is over-oxidized and has a hydrate layer separating the iodate oxidizer from Al° fuel that dehydrates at 140 °C. When the hydrate layer is evaporated, excess oxygen from AIH reacts with Al° without the diffusion limitations of the Al_2_O_3_ passivation layer. The improved laser-induced shock velocities in Fig. 4 show that Al particles coated with AIH react on timescales relevant to detonation waves. Further optimization of AIH synthesis to tailor AIH mixtures could result in additional increases in explosive performance.

## Conclusions

Aluminum particles have been synthesized with an AIH coating. The crystalline AIH replaces the Al_2_O_3_ shell encapsulating an Al core particle and is observed using TEM analysis as well as detected in powder XRD analysis. Two samples were synthesized, the first contained 5.8% AIH on Al (i.e., AIH6) and the second contained 15% AIH on Al (i.e., AIH15).

The energy release of both samples was examined via LASEM testing. The technique enables analysis of small quantities (~mg) of material in order to assess the feasibility of larger scale-up efforts that evaluate detonation performance. The LASEM results for the AlH samples demonstrated their potential use for explosive enhancement on both fast (detonation velocity) and slow (blast effects) timescales. Estimates of the detonation velocity for TNT-AIH composites suggest an enhancement of up to 30% may be achievable over pure TNT detonation velocities. The laser-induced deflagrations of the TNT-AIH composites also suggest the potential for blast enhancement effects.

The mechanisms for AIH-Al coated particles contributing to the early-time reactions may include: enhanced reaction of the under-oxidized TNT (OB of −74%) with the over-oxidized AIH and the chemical structure of AIH, which includes a hydrate layer separating the iodate oxidizer from Al^0^ fuel that dehydrates at low temperatures, 140 °C. In this way, AIH reacts with Al^0^ without the diffusion limitations of the Al_2_O_3_ passivation layer. Gas production via dehydration and exothermic reactions may also increase the temperature of the plasma therefore increasing the shock velocity. Unreacted Al remaining after the passage of the shock front may then provide the enhanced blast effects at later times without the hindrance of the Al_2_O_3_ passivation layer inherent in Al particles.

While the interpretation of these preliminary results are complicated by the complexity of the laser-material interaction and subsequent shot-to-shot variations, we believe these results suggest larger-scale detonation tests are worth pursuing, assuming the composite materials pass the prerequisite safety and compatibility testing prior to scale-up (including sensitivity testing). The degradation of the AIH samples under atmospheric conditions must also be sufficiently addressed for viability in military applications.

### Data Availability Statement

Data is available in the supplementary information section.

## Electronic supplementary material

Supplementary Information

## References

[1] Gottfried JL (2012). Laser-Induced Plasma Chemistry of the Explosive RDX with Various Metallic Nanoparticles. Appl. Opt..

[2] Gottfried JL (2014). Influence of Exothermic Chemical Reactions on Laser-Induced Shock Waves. Phys. Chem. Chem. Phys..

[3] Gottfried JL (2015). Laboratory-Scale Method for Estimating Explosive Performance from Laser-Induced Shock Waves. Propellants, Explos. Pyrotech..

[4] Fischer D (2016). Synthesis and Investigation of Advanced Energetic Materials Based on Bispyrazolylmethanes. Angew. Chemie..

[5] Gottfried JL, Witkowski TG, Klapotke TM (2017). Estimated Detonation Velocities for TKX-50, MAD-X1, BDNAPM, BTNPM, TKX-55, and DAAF Using the Laser – Induced Air Shock from Energetic Materials Technique. Propellants, Explos. Pyrotech..

[6] Gottfried JL, Bukoswski EJ (2017). Laser-Shocked Energetic Materials with Metal Additives: Evaluation of Chemistry and Detonation Performance. Appl. Opt..

[7] Collins ES, Gottfried JL (2017). Laser-Induced Deflagration for the Characterization of Energetic Materials. Propellants, Explos. Pyrotech..

[8] Mcnesby KL (2010). Afterburn Ignition Delay and Shock Augmentation in Fuel Rich Solid Explosives. Propellants, Explos. Pyrotech..

[9] Capellos C (2007). Eigenvalue Detonation of Combined Effects Aluminized Explosives. AIP Conference Proceedings.

[10] Baker, E. L., Stiel, L., Balas, W., Capellos, C. & Pincay, J. Combined Effects Aluminized Explosives. I*nt*. *Symp*. *Ballist*., *Proc*. *24*^*th*^, 2 (July), 1135–1143 (2008).

[11] Cradwick PD, Endredy AS (1977). Crystal Structre of Aluminium Iodate-Hydrogen Iodate-Water (1/1/6) and Preparation of Anhydrous Aluminium Iodate. J. Chem. Soc..

[12] Smith DK, Bello MN, Unruh DK, Pantoya ML (2017). Synthesis and Reactive Characterization of Aluminum Iodate Hexahydrate Crystals [Al(H_2_O)6](IO_3_)_3_(HIO_3_)_2_. Combust. Flame..

[13] Smith DK, Unruh DK, Pantoya ML (2017). Replacing the Al_2_O_3_ Passivation Shell of Al Particles with an Energetic Salt: AIH. Part 2, Synthesis. J. Phys. Chem. C..

[14] Smith DK, Unruh DK, Pantoya ML (2017). Replacing the Al_2_O_3_ Passivation Shell of Al Particles with an Energetic Salt: AIH. Part 1: Reactivity. J. Phys. Chem. C..

[15] Ayache, J., Beaunier, L., Boumendil, J., Ehret, Gabrielle, E. & Laub, D. *Sample Preparation Handbook for Transmission Electron Microscopy Techniques*, (Springer, 2009).

[16] Russo RE, Mao X, Mao SS (2002). The Physics of Laser Ablation in Microchemical Analysis. Anal. Chem..

[17] Bogaerts A, Chen Z (2005). Effect of Laser Parameters on Laser Ablation and Laser-Induced Plasma Formation: A Numerical Modeling Investigation. Spectrochim. Acta- Part B At. Spectrosc..

[18] Michel APM, Chave AD (2007). Analysis of Laser-Induced Breakdown Spectroscopy Spectra: The Case for Extreme Value Statistics. Spectrochim Acta Part B..

[19] Gottfried JL, De Lucia FCJ, Munson CA, Miziolek AW (2008). Strategies for Residue Explosives Detection Using Laser-Induced BreakdownSpectroscopy. J. Anal. At. Spectrom..

[20] Tognoni E, Cristoforetti G, Legnaioli S, Palleschi V (2010). Laser-Induced Breakdown Spectroscopy: State of the Art. Spectrochim. Acta Part B At. Spectrosc..

[21] Gottfried, J. L. Chemometric Analysis in LIBS, in *Handbook of Laser-Induced Breakdown Spectroscopy*, 2nd ed., (eds. Cremers, D. A., Radziemski, L. J.) (John Wiley and Sons Inc.: Singapore, 2013).

[22] Martin JCG, Spietz P, Burrows JP (2007). Kinetic and Mechanistic Studies of the I2_/_O3 Photochemistry. J. Phys. Chem. A..

