# Supplementary material for: Improving the Explosive Performance of Aluminum Nanoparticles with Aluminum Iodate Hexahydrate (AIH)
Source: Sci Rep. 2018 May 23;8:8036. doi: 10.1038/s41598-018-26390-9 (PMC5966432; doi:10.1038/s41598-018-26390-9)
Supplement: Supplementary file 1 — Supplementary Information [file 41598_2018_26390_MOESM1_ESM.docx]

**Improving the Explosive Performance of Aluminum Nanoparticles with Aluminum Iodate Hexahydrate (AIH)**

Jennifer L. Gottfried^2^, Dylan K. Smith^1^, Chi-Chin Wu^2^, Michelle L. Pantoya^1*^

^1^Department of Mechanical Engineering, Texas Tech University, Lubbock, TX 79409

^2^Weapons and Materials Research Directorate, U.S. Army Research Laboratory, Aberdeen Proving Ground, Aberdeen, MD, 21005

*Corresponding author contact information: Phone: 806-834-3733; email: [michelle.pantoya@ttu.edu](mailto:michelle.pantoya@ttu.edu)

Supplemental Figures


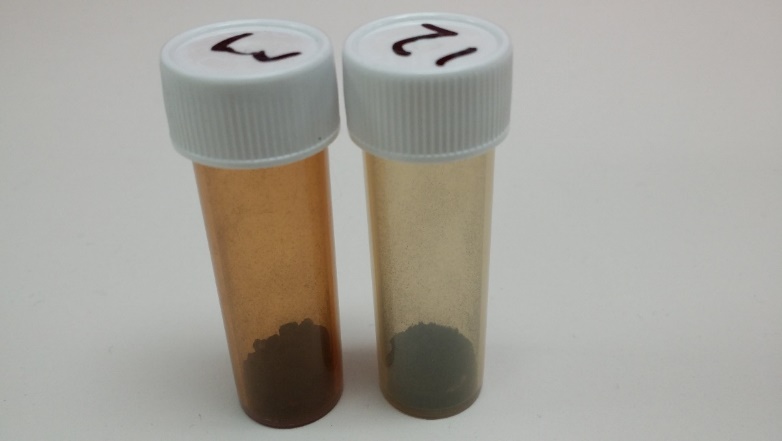


Figure S1. Photograph of aluminum iodate hexahydrate (AIH) samples AIH6 (left) and AIH15 (right) as received for LASEM analysis. See Table 1 for full chemical composition of these samples.


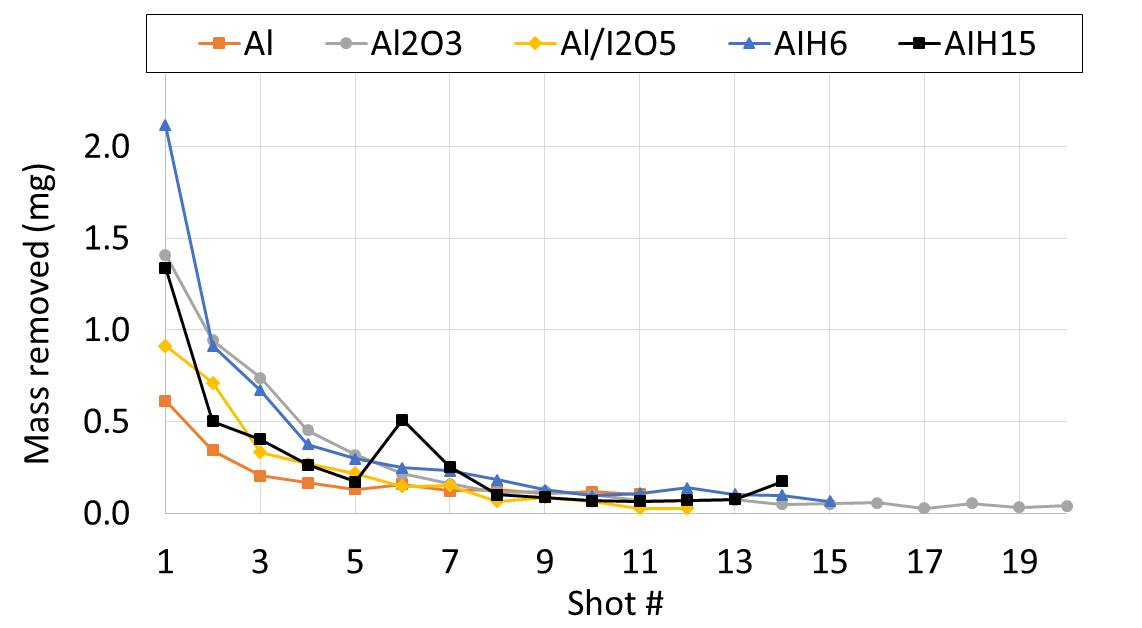


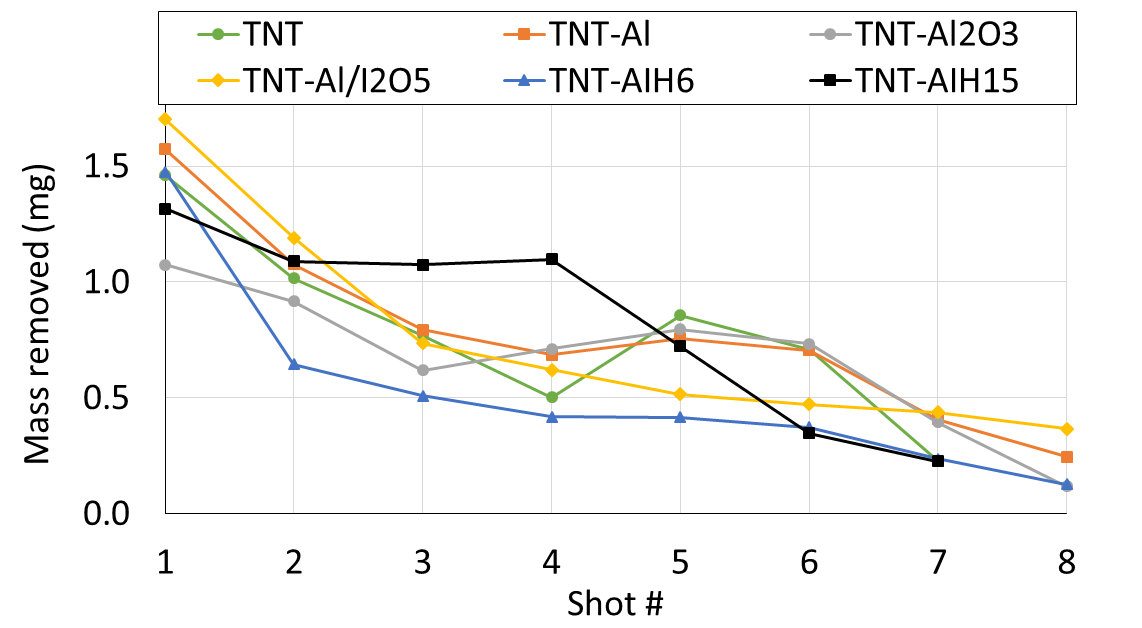


Figure S2.Mass of material removed per laser shot from pure (top) and composite (bottom) samples.


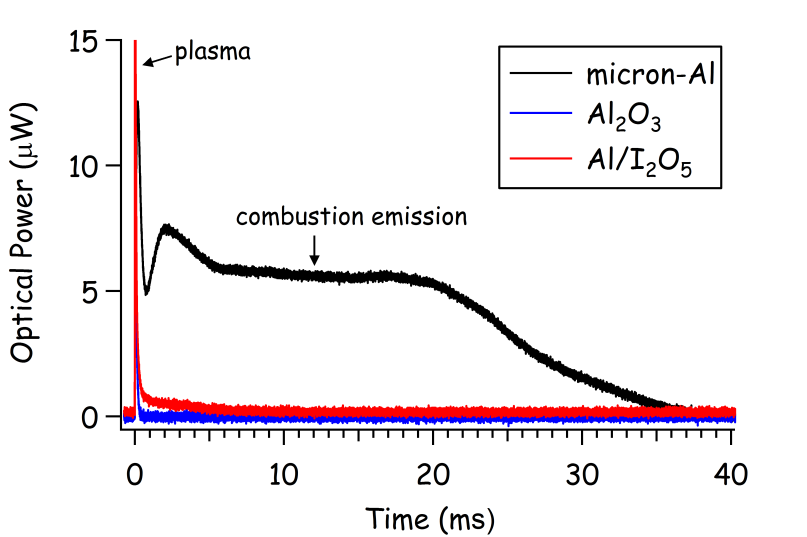


Figure S3. Average time-resolved emission for micron-sized Al (black), Al_2_O_3_ (blue), and Al/I_2_O_5_ (red) samples following laser ablation.


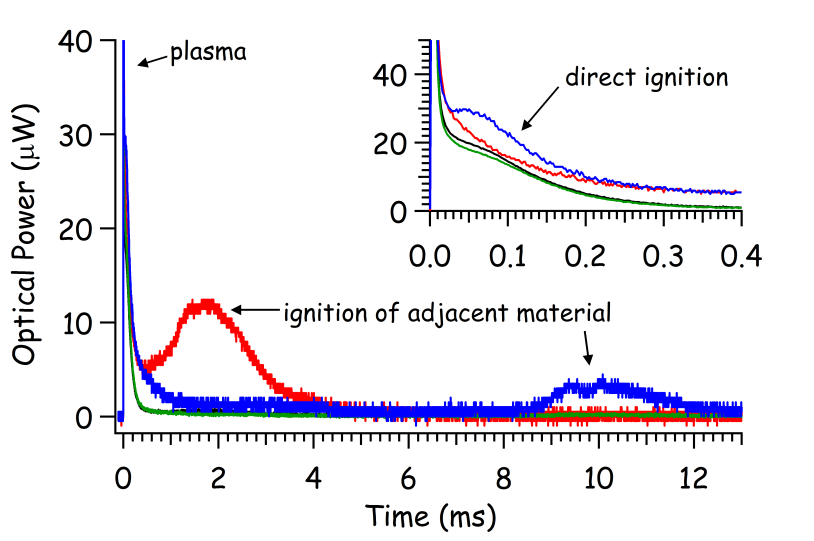


Figure S4. Average (green, black) and single-shot (blue, red) time-resolved emission for AIH6 and AIH15, respectively; the first 2 laser shots on each of the sample slides resulted in ignition of material adjacent to the laser focal position.


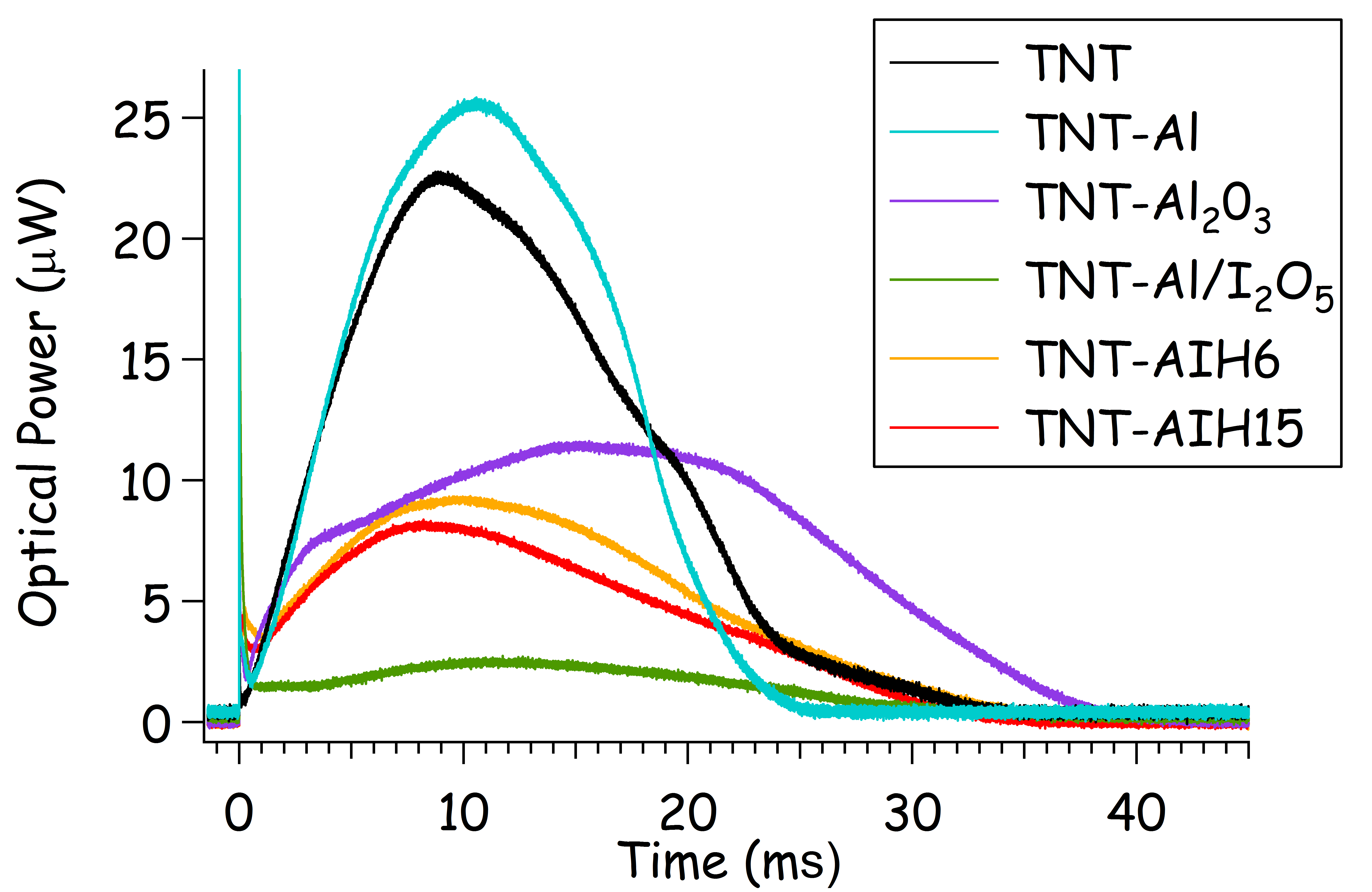


*Figure S5. Average time-resolved emission from the laser-induced deflagration of TNT composites (all laser shots).*


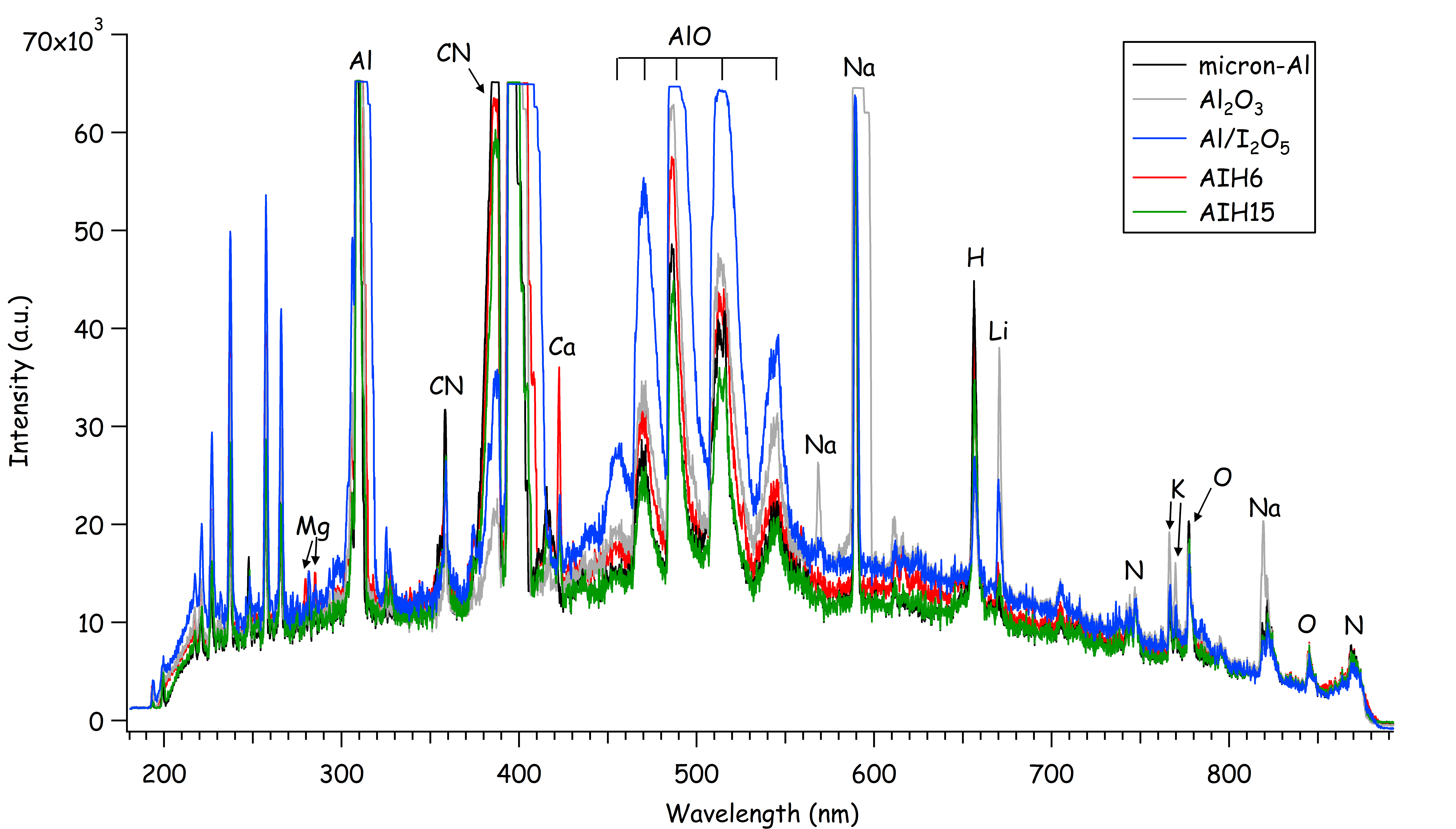


Figure S6. Emission spectra of metal additives; unlabeled strong emission lines below 350 nm resulted from atomic or singly ionized Al.


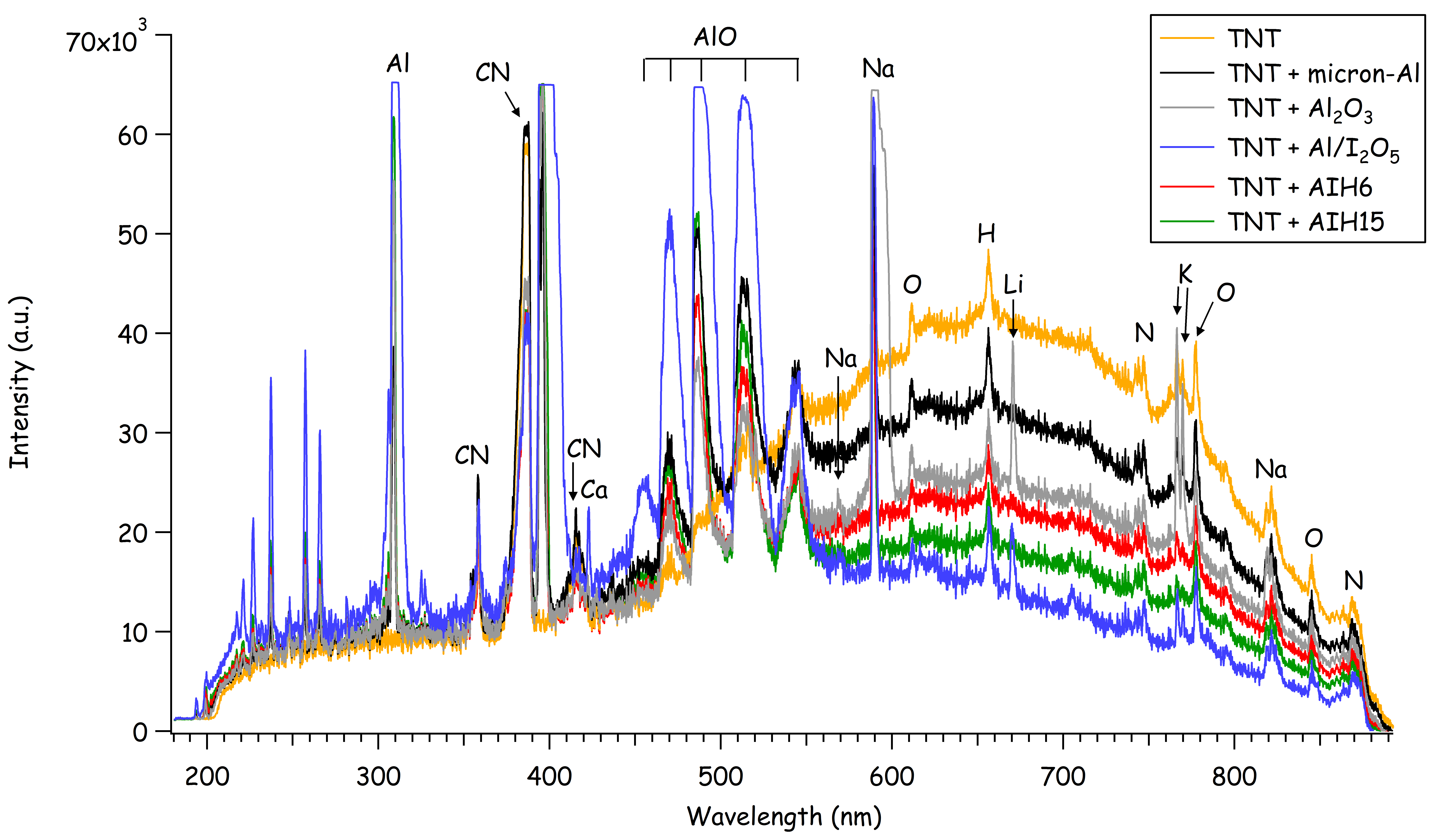


Figure S7. Emission spectra of TNT and TNT with metal additives; unlabeled strong emission lines below 350 nm resulted from atomic and singly ionized Al.
